# Supplementary material for: HIVprotI: an integrated web based platform for prediction and design of HIV proteins inhibitors
Source: J Cheminform. 2018 Mar 9;10:12. doi: 10.1186/s13321-018-0266-y (PMC5845081; doi:10.1186/s13321-018-0266-y)
Supplement: Supplementary file 2 — Additional file 2. Source code of HIVProtI web server. [file 13321_2018_266_MOESM2_ESM.zip › HIVprotI_Source-code/hivprotI/weka-3-6-6/doc/weka/gui/BrowserHelper.html]

BrowserHelper


---


|  |  |  |
| --- | --- | --- |
| **Overview** \| **Package** \| **Class** \| **Tree** \| **Deprecated** \| **Index** \| **Help** \| **Weka's home** | |  |
| **PREV CLASS**   **NEXT CLASS** | **FRAMES**    **NO FRAMES**     **All Classes** |  |
| SUMMARY: NESTED \| FIELD \| CONSTR \| METHOD | DETAIL: FIELD \| CONSTR \| METHOD |  |


---


## weka.gui Class BrowserHelper

```
java.lang.Object
  weka.gui.BrowserHelper
```

---

``` public class BrowserHelper extends java.lang.Object ```

A little helper class for browser related stuff.

The `openURL` method is based on
Bare Bones Browser Launch,
which is placed in the public domain.

**Version:**
:   $Revision: 7059 $

**Author:**
:   fracpete (fracpete at waikato dot ac dot nz)

---

| **Field Summary** | |
| --- | --- |
| `static java.lang.String[]` | `LINUX_BROWSERS`             Linux/Unix binaries to look for |


**Constructor Summary**

`BrowserHelper()`


| **Method Summary** | |
| --- | --- |
| `static void` | `openURL(java.awt.Component parent, java.lang.String url)`             opens the URL in a browser. |
| `static void` | `openURL(java.awt.Component parent, java.lang.String url, boolean showDialog)`             opens the URL in a browser. |
| `static void` | `openURL(java.lang.String url)`             opens the URL in a browser. |

**Methods inherited from class java.lang.Object**

`equals, getClass, hashCode, notify, notifyAll, toString, wait, wait, wait`

**Field Detail**

### LINUX\_BROWSERS

```
public static final java.lang.String[] LINUX_BROWSERS
```

:   Linux/Unix binaries to look for


**Constructor Detail**

### BrowserHelper

```
public BrowserHelper()
```


**Method Detail**

### openURL

```
public static void openURL(java.lang.String url)
```

:   opens the URL in a browser.

    :   **Parameters:**: `url` - the URL to open

---


### openURL

```
public static void openURL(java.awt.Component parent,
                           java.lang.String url)
```

:   opens the URL in a browser.

    :   **Parameters:**: `parent` - the parent component: `url` - the URL to open

---


### openURL

```
public static void openURL(java.awt.Component parent,
                           java.lang.String url,
                           boolean showDialog)
```

:   opens the URL in a browser.

    :   **Parameters:**: `parent` - the parent component: `url` - the URL to open: `showDialog` - whether to display a dialog in case of an error or just print the error to the console


---


|  |  |  |
| --- | --- | --- |
| **Overview** \| **Package** \| **Class** \| **Tree** \| **Deprecated** \| **Index** \| **Help** \| **Weka's home** | |  |
| **PREV CLASS**   **NEXT CLASS** | **FRAMES**    **NO FRAMES**     **All Classes** |  |
| SUMMARY: NESTED \| FIELD \| CONSTR \| METHOD | DETAIL: FIELD \| CONSTR \| METHOD |  |


---
